# Practical Chemistry for Medical Students

**Published:** 1892-06

**Authors:** 


*Practical Chemistry for Medical Students.* By SAMUEL RIDEAL, D.Sc. Lond. Pp. 55. London: H. K. LEWIS. 1890.

A medical student, who has to present himself for examination under the conjoint board, will find in this small book instructions for testing those simple substances which he would then be required to identify. The details here given would, if mastered, surely create an appetite for more.

